# Associations of Fentanyl, Sufentanil, and Remifentanil With Length of Stay and Mortality Among Mechanically Ventilated Patients: A Registry-Based Cohort Study

**DOI:** 10.3389/fphar.2022.858531

**Published:** 2022-03-04

**Authors:** Wen Wang, Qiao He, Mingqi Wang, Yan Kang, Peng Ji, Shichao Zhu, Rui Zhang, Kang Zou, Xin Sun

**Affiliations:** ^1^ Chinese Evidence-based Medicine Center, West China Hospital, Sichuan University, Chengdu, China; ^2^ NMPA Key Laboratory for Real World Data Research and Evaluation in Hainan, Chengdu, China; ^3^ Intensive Care Unit, West China Hospital, Sichuan University, Chengdu, China; ^4^ Department of Infection Control, West China Hospital, Sichuan University, Chengdu, China; ^5^ Information Center, West China Hospital, Sichuan University, Chengdu, China

**Keywords:** mechanical ventilation, intensive care unit, fentanyl, sufentanil, remifentanil

## Abstract

**Background:** As the first-line treatment for mechanically ventilated patients with critical illness, fentanyl and its analogs (e.g., sufentanil and remifentanil) are commonly used in the intensive care unit (ICU). However, the pharmacokinetics, metabolism, and potency of these agents differed. Their effects on clinical outcomes have not been well-understood.

**Materials and Methods:** Using a well-established registry, we conducted a cohort study. Patients who consistently underwent mechanical ventilation (MV) for more than 24 h were identified. We used a time-varying exposure definition, in which we coded each type of opioids as prescribed or not prescribed on each day from initiation of MV to extubation and ICU discharge. We used Fine-Gray competing risk models to compare the effects of fentanyl, sufentanil, and remifentanil on hazards for extubation, ventilator mortality, ICU discharge, and ICU mortality. All models were adjusted using a combination of fixed-time and time-varying covariates. Missing data were imputed using multiple imputation by chained equations.

**Results:** A total of 8,165 patients were included. There were, respectively, 4,778, 4,008, and 2,233 patients receiving at least 1 day of fentanyl, sufentanil, and remifentanil dose. Compared to fentanyl, sufentanil was associated with shorter duration to extubation (hazard ratio 1.31, 95% CI, 1.20–1.41) and ICU discharge (hazard ratio 1.63, 95% CI, 1.38–1.92), and remifentanil was associated with shorter duration to extubation (hazard ratio 1.60, 95% CI, 1.40–1.84) and ICU discharge (hazard ratio 2.02, 95% CI, 1.43–2.84). No significant differences in time to extubation (Hazard ratio 1.14, 95% CI, 0.92–1.41) and ICU discharge (Hazard ratio 1.31, 95% CI, 0.81–2.14) were found between sufentanil and remifentanil. No differences were observed between any two of the agents regarding ventilator mortality or ICU mortality. The effects were similar in patients with versus without surgery.

**Conclusion:** Sufentanil and remifentanil may be superior to fentanyl in shortening the time to extubation and ICU discharge. The effects on ventilator mortality and ICU mortality appeared similar across these agents, while further research is warranted.

## Introduction

Pain is common among patients admitted to the intensive care unit (ICU) (Jeitziner et al., 2012; Ehieli et al., 2017). Intravenous opioids are usually recommended as the first-line treatment for non-neuropathic pain among patients with critical illness, especially for those receiving mechanical ventilation (MV) (Faust et al., 2016; Devlin et al., 2018; Jungquist et al., 2019). Previous studies showed that 90% of patients received opioids during MV treatment, and fentanyl and fentanyl analogs were the most commonly prescribed opioids; about 75–85% of patients received fentanyl, sufentanil, and remifentanil treatment during MV (Payen et al., 2007).

Although widely used, these opioid agents vary in their pharmacokinetic characteristics and risk of accumulation in organ failure (Ehieli et al., 2017; Zhu et al., 2017; Armenian et al., 2018). For instance, remifentanil is metabolized by unspecific esterases independent of liver or renal function, and the duration of action (5–10 min) is shorter relative to fentanyl and sufentanil. While sufentanil is 5–10 times potent than fentanyl and remifentanil, the risk of opioid accumulation in organs may be higher than that of remifentanil (Maciejewski, 2012). The accumulation of opioids may cause respiratory depression, sedation, hypotension, and immunosuppression, which may further result in adverse outcomes (Zhu et al., 2017; Devlin et al., 2018; Gupta et al., 2018).

The pharmacokinetic differences among these agents may lead to differential patient-important clinical outcomes, such as respiratory depression and death. However, evidence regarding the effects of these agents on ICU patients receiving MV is inadequate. Several randomized controlled trials (RCTs) investigated the effects of these agents, but were small in sample sizes and had inconsistent findings.(Breen et al., 2005; Djian et al., 2006; Rozendaal et al., 2009; Spies et al., 2011; Bhavsar et al., 2016; Zhu et al., 2017). One meta-analysis involving 1905 mechanically ventilated patients compared the effectiveness of analgesic regimens with versus without remifentanil; however, this study compared remifentanil versus all other opioids, leaving the comparison among fentanyl and its analogs an unanswered question (Zhu et al., 2017). One additional important issue that existing studies did not address is how varying exposure to opioids during MV treatment in routine practice may have affected treatment outcomes (Klompas et al., 2016b).

Therefore, we conducted a cohort study to compare the effects of fentanyl, sufentanil, and remifentanil on extubation, ICU discharge, and ICU mortality. This study included a large number of patients and used statistical methods to handle daily change in the exposure to fentanyl and its analogs.

## Materials and Methods

### Data Source

This cohort study was conducted using a registry of healthcare-associated infections (HAIs) at ICUs from the West China Hospital (WCH) system (Wang et al., 2019). The WCH system includes three independent healthcare organizations and contains six ICUs, and is a critical care center that covers population in West China. Every year, there were more than 200,000 inpatient visits, and 8,000 patients were admitted to ICUs.

The registry was established by integrating three databases, including the electronic medical record (EMR) system, ICU system, and ICU-HAI system. All patients admitted to any of six ICUs since April 1, 2015 were included in the registry. Till December, 2018, a total of 29,480 ICU admissions were included. Detailed information about the registry has been published elsewhere (Wang et al., 2019). The registry has a high level of quality and comprehensiveness of information and has been utilized for addressing various clinical research questions(Zhu et al., 2019; Wang et al., 2021a; Wang et al., 2021b; He et al., 2021; Zhu et al., 2021). Quality assessment showed that the accuracy of data extraction and linkage was 100%, and the completeness of important laboratory tests was more than 98%. The International Classification of Diseases 10th Revision (ICD-10) codes at WCH have been validated, and the completeness and accuracy were 99 and 88%, respectively (Wang et al., 2019; Wang et al., 2021b).

This study was approved by the West China Hospital Institutional Review Board (WCH 2018–409), who granted a waiver of patient consent.

### Study Population

The patients were eligible in the cohort if they consistently received MV for more than 24 h between April 1, 2015 and December 31, 2018. The patients were excluded if they met any of the following criteria: patients with incomplete information including date of birth, sex, and discharged diagnosis; age < 18 years or admitted to the pediatric ICU; without receiving any opioids during ICU stay. The clinical characteristics differed among patients with and without extremely long ICU stay (>90 days). To minimize indication bias, we also excluded patients with extremely long ICU stay. If patients experienced multiple episodes of MV, we only measured the first episode of MV treatment for more than 24 h.

### Drug Exposure and Outcomes

The exposures of interest included fentanyl, sufentanil, and remifentanil. We used a time-varying exposure definition. Information regarding daily exposures to fentanyl, sufentanil, and remifentanil was extracted from the prescription data of the EMR system. We coded each type of opioids (fentanyl, sufentanil, and remifentanil) as prescribed or not prescribed on each day from initiation of MV to extubation and each day from initiation of MV to ICU discharge, respectively.

The outcomes included duration of MV, ventilator mortality, length of ICU stay, and ICU mortality, which were measured as time-to-event variables. Patients who died within 1 day after extubation and 1 day after ICU discharge were defined as ventilator mortality and ICU mortality, respectively.

### Confounding Factors

We identified potential confounding factors based on clinical plausibility. We also consulted experts in critical care medicine, anesthesia, and respiratory therapy to identify potential confounding factors. The potential confounding factors included demographic characteristics (age, sex, and ICU type), acute conditions at ICU admission (acute respiratory distress syndrome (ARDS), shock, gastrointestinal bleeding, and pneumonia), chronicity comorbidities (diabetes, malignant tumor, chronic lung disease, cardiovascular disease, heart failure, liver and renal failure, and hypertension), APACHE II score, fluid balance, surgery before the event of interest (thoracic surgery, cranial surgery, cardiac surgery, and abdominal surgery), fiberoptic bronchoscopy examination, tracheotomy, concomitant medications (non-opioid analgesics and other types of opioids (e.g., morphine, pethidine), sedatives, neuroleptic agents, thromboembolism agents, acid inhibitors, neuromuscular blockers, intestinal probiotics, expectorants, antibiotics, immunosuppressive agents, and vasopressors), and other treatment (blood transfusion, gastrointestinal decompression, prone position ventilation, enteral nutrition, mandatory ventilation, esophagogastroduodenoscopy, and head-of-bed elevation). We also included days from ICU admission to initiation of MV, and opioids prescribed before the initiation of MV as potential confounding factors.

We identified concomitant medications from prescription data and comorbidity from discharge diagnoses using ICD-10 codes.

### Statistical Analysis

We assessed the impact of daily fentanyl, sufentanil, and remifentanil exposures on time to extubation, ventilator mortality, time to ICU discharge, and ICU mortality using Fine-Gray competing risk models. Fine-Gray competing risks models are proportional subdistribution hazard models which can disentangle effects of competing risks and outcomes of interest by generating separate hazard ratios for each competing event (Mohammad et al., 2017). The reason for extubation and ICU discharge varies considerably depending on the patient’s health condition: clinical improvement versus death. Therefore, we used Fine-Gray competing risk models to measure the competing risks for extubation alive versus ventilator mortality and ICU discharge alive versus ICU mortality.(Klompas et al., 2016a; Klompas et al., 2016b). We calculated hazard ratios (HRs) for sufentanil versus fentanyl, remifentanil versus fentanyl, and sufentanil versus remifentanil.

All models were adjusted using a combination of fixed-time and time-varying covariates. We defined demographic characteristics, acute conditions, chronicity comorbidities, APACHE II score, surgery, fiberoptic bronchoscopy examination, tracheotomy, days from ICU admission to initiation of MV, and opioids prescribed before the initiation of MV as fixed-time variables. We defined exposures to concomitant medications and other treatment as time-varying variables. We measured time-varying variables as daily exposure on each day of MV treatment for the model of extubation alive versus ventilator mortality and each day from initiation of MV to ICU discharge for the model of ICU discharge versus ICU death. We additionally adjusted the duration of mechanical ventilation and measured mandatory ventilation and prone position ventilation as fixed-time variables for the analysis of ICU discharge versus ICU death.

The percentages of missing data for APACHE II score and fluid balance during ICU stay were 9.18 and 8.68%, respectively. We handled missing data of APACHE II score and fluid balance using multiple imputation by chained equations (MICE), and the iterations were set to five times. By creating a number of datasets to handle uncertainty in missing value imputation, multiple imputation is thought to be superior to single imputation and complete-case analysis (Zhang, 2016).

### Subgroup Analyses and Sensitivity Analyses

We calculated HRs for patients with versus without receiving surgery separately, given that the clinical characteristics and treatment approach differed variously between these two populations.

We performed several sensitivity analyses to test the robustness of the primary findings. First, we used the traditional Cox model with time-varying covariates to calculate the effect estimates. Second, we used complete case analysis without imputation to handle missing data. Third, to address the potential confounding factors by indication, we restricted to patients who did not receive non-opioid analgesics. All analyses were performed using R version 3·6·1.

## Results

**FIGURE 1:**
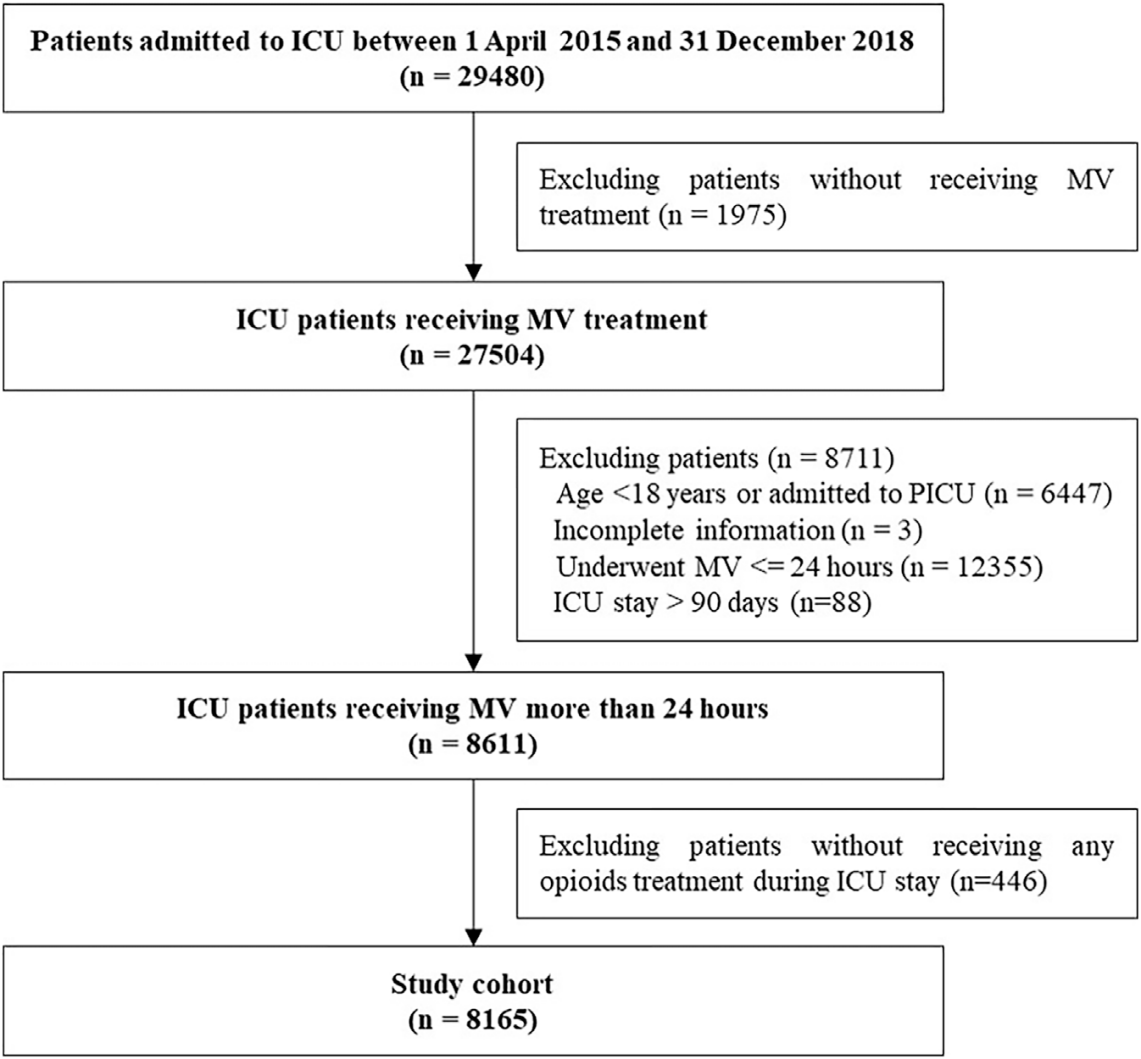
Study flow chart.

The study included 8,165 patients who consistently received MV for at least 24 h and treated with opioids during ICU stays from April 1, 2015 to December 31, 2018. Of 8,165 included patients, 4,778 received at least 1 day of fentanyl, 4,008 received at least 1 day of sufentanil, and 2,233 received at least 1 day of remifentanil (see Figure 1).

Among all included patients, the median age was 57 (interquartile range (IQR) years, 46–68); 3,147 (38.5%) patients were females; the median APACHE II score was 19 (IQR, 14–24). There were 2,457 (30.1%) patients admitted to the general ICU and 1705 (20.9%) admitted to the surgical ICU. The most common comorbidities were hypertension (21.2%), heart failure (12.5%), and chronic lung disease (9.8%), and the most common acute conditions were pneumonia (7.7%) and shock (5.4%). There were 1,444 (17.7%) patients who underwent cardiac surgery, 809 (9.9%) who underwent thoracic surgery, 1,465 (17.9%) who underwent cranial surgery, and 1,573 (19.3%) who underwent abdominal surgery. The clinical characteristics differed among patients receiving different opioids. For instance, the proportions of cardiac surgery were 4.7% among patients receiving fentanyl and 24.7% among patients receiving remifentanil (see Table 1).

**TABLE 1:** Clinical characteristics of included patients.

Characteristics	Overall patients (*n* = 8,165)	Fentanyl (*n* = 4,778)	Sufentanil (*n* = 4,008)	Remifentanil (*n* = 2,233)
Age, median [IQR]	57 [46, 68]	58.00 [45, 70]	56 [45, 67]	53.00 [44, 65]
Sex, female (%)	3,147 (38.5)	1,696 (35.5)	1,524 (38.0)	915 (41.0)
APACHE II, median [IQR]	19 [14, 24]	20 [15, 25]	19 [14, 23]	18 [13, 22]
ICU type (%)				
General ICU	2,457 (30.1)	1712 (35.8)	1,557 (38.8)	472 (21.1)
Neurological ICU	1,338 (16.4)	1,080 (22.6)	423 (10.6)	412 (18.5)
Respiratory ICU	1,139 (13.9)	1,074 (22.5)	384 (9.6)	92 (4.1)
Surgical ICU	1705 (20.9)	675 (14.1)	957 (23.9)	692 (31.0)
Thoracic ICU	1,526 (18.7)	237 (5.0)	687 (17.1)	565 (25.3)
Acute conditions (%)				
ARDS	76 (0.9)	60 (1.3)	46 (1.1)	14 (0.6)
Shock	441 (5.4)	290 (6.1)	302 (7.5)	150 (6.7)
Gastrointestinal bleeding	112 (1.4)	81 (1.7)	66 (1.6)	25 (1.1)
Pneumonia	632 (7.7)	433 (9.1)	325 (8.1)	121 (5.4)
Comorbidities (%)				
Diabetes	646 (7.9)	399 (8.4)	293 (7.3)	147 (6.6)
Cardiovascular disease	148 (1.8)	63 (1.3)	49 (1.2)	30 (1.3)
Heart failure	1,020 (12.5)	250 (5.2)	467 (11.7)	341 (15.3)
Chronic lung disease	798 (9.8)	640 (13.4)	334 (8.3)	94 (4.2)
Malignant tumor	676 (8.3)	408 (8.5)	366 (9.1)	140 (6.3)
Liver failure	158 (1.9)	112 (2.3)	81 (2.0)	25 (1.1)
Hypertension	1734 (21.2)	961 (20.1)	798 (19.9)	516 (23.1)
Kidney failure	485 (5.9)	350 (7.3)	216 (5.4)	79 (3.5)
Surgery (%)				
Cardiac surgery	1,444 (17.7)	226 (4.7)	672 (16.8)	552 (24.7)
Thoracic surgery	809 (9.9)	560 (11.7)	432 (10.8)	168 (7.5)
Cranial surgery	1,465 (17.9)	927 (19.4)	754 (18.8)	656 (29.4)
Abdominal surgery	1,573 (19.3)	871 (18.2)	1,117 (27.9)	621 (27.8)
Outcomes				
Days of MV, median (IQR)	5 [3, 11]	7 [4, 13]	5 [3, 11]	5 [2, 9]
ICU stays, median (IQR)	10 [5, 19]	13 [7, 22]	11 [6, 21]	9 [5, 19]
ICU mortality (%)	971 (11.9)	742 (15.5)	446 (11.1)	161 (7.2)

Abbreviations: IQR, interquartile range; ICU, intensive care unit; ARDS, acute respiratory distress syndrome; MV, mechanical ventilation.

Among all included patients, the median day of MV was 5 (IQR, 3–11), the median ICU stay was 10 (IQR, 5–19) days, and crude ICU mortality was 11.9%. Among patients receiving fentanyl, sufentanil, and remifentanil, the median ICU stay was 13 (IQR, 7–22), 11 (IQR, 6–21) and 9 (IQR, 5–19) days; the crude mortality was 15.5, 11.1, and 7.2% (see Table 1).

### Opioid Treatment Among Included Patients

**FIGURE 2:**
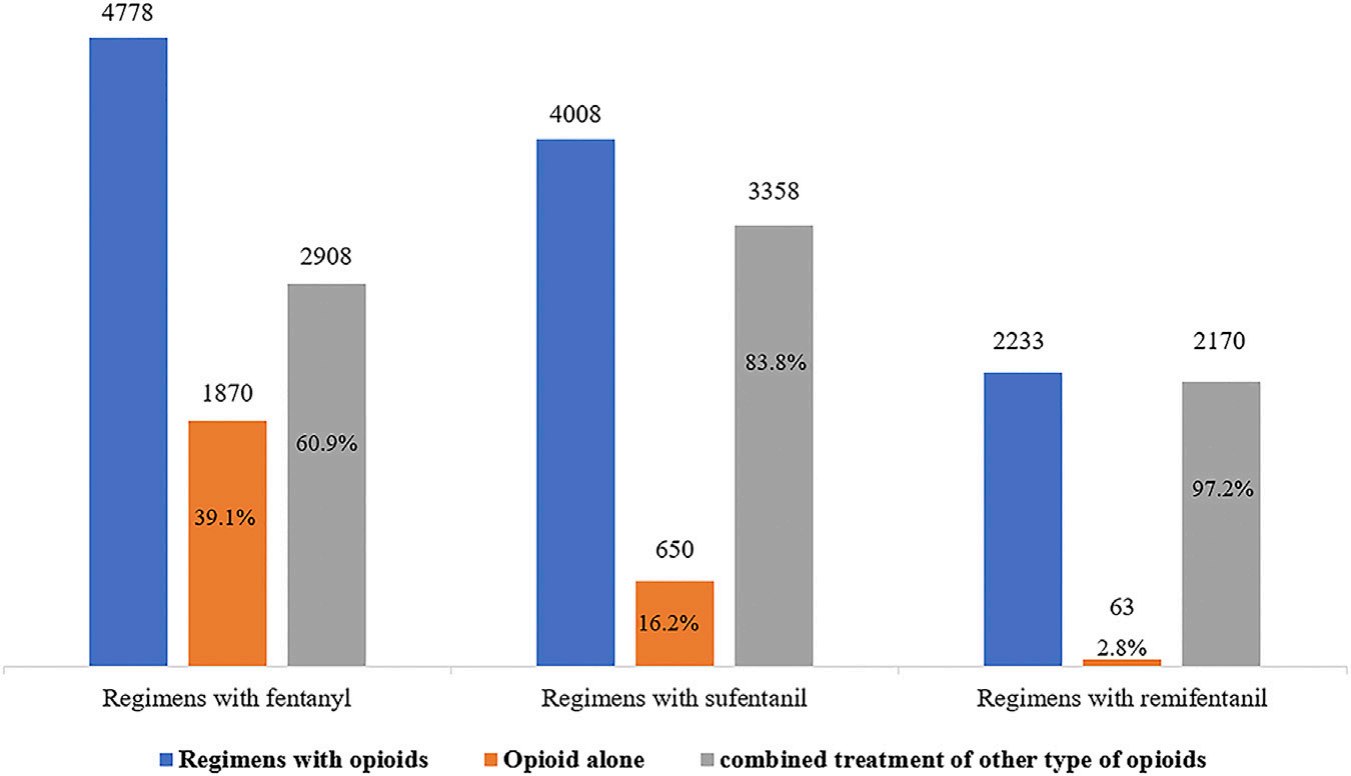
Treatment of opioids during ICU stay.

Opioids were often used concurrently in patients receiving MV. Among patients receiving fentanyl, sufentanil, and remifentanil, the proportion of patients treated with other opioids during ICU stay was 60.9, 83.8, and 97.2%, respectively (see Figure 2). Moreover, combined treatment of opioids with sedatives was common; the proportion among patients receiving fentanyl, sufentanil, and remifentanil was 74.9, 72.6, and 70.4%, respectively.

The median cumulative dose fentanyl, sufentanil and remifentanil administrated during ICU stays was 4.5 mg (IQR, 1.5–10), 0.4 mg (IQR, 0.14–1.1) and 1.0 mg (IQR, 1.0–2.0), respectively. The median days of use of fentanyl [4 days (IQR, 2–9)] was longer than sufentanil [1 day (IQR, 1–5)] and remifentanil [1 day (IQR, 1–1)] (See Supplementary Table S1).

### Associations Between Opioid Exposures and Outcomes

**FIGURE 3:**
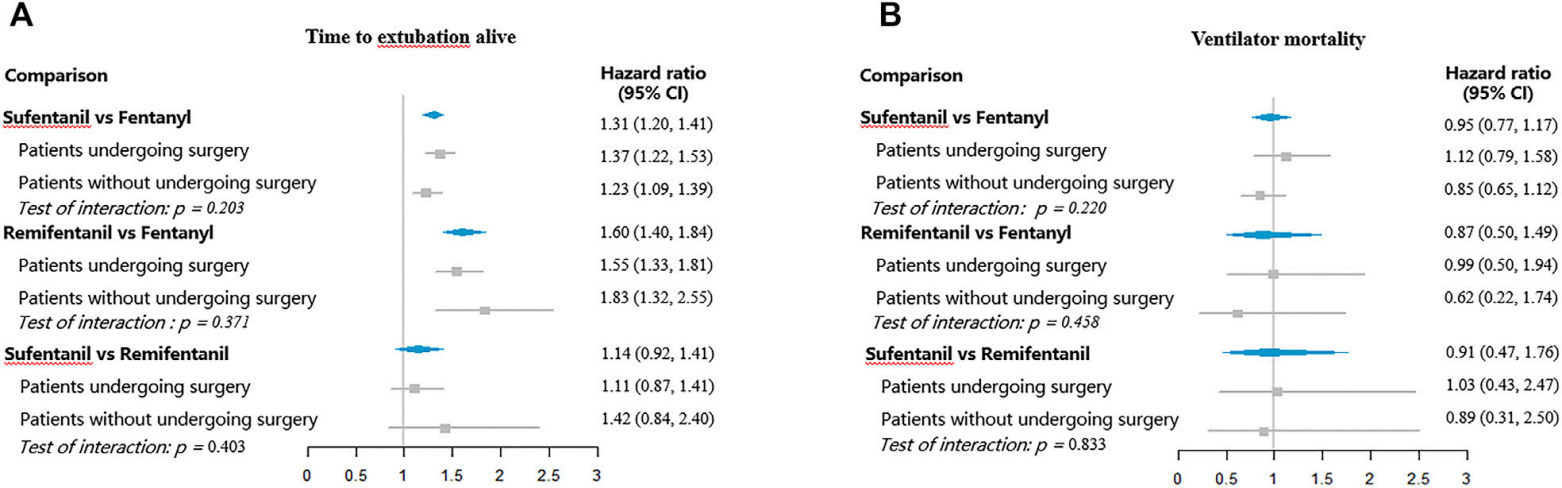
Hazard ratios for extubation and ventilator mortality with different opioids. **(A)** Time to extubation alive; **(B)** Ventilator mortality.

**FIGURE 4:**
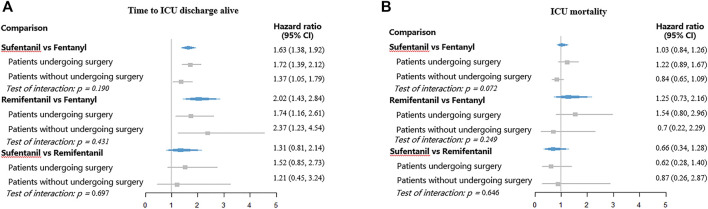
Hazard ratios for ICU discharge and ICU mortality with different opioids. **(A)** Time to ICU discharge alive; **(B)** ICU mortality.

The adjusted estimates of opioids on the predefined clinical outcomes are shown in Figures 3, 4. Compared to fentanyl, sufentanil was associated with an increased hazard for extubation (HR 1.31, 95% CI, 1.20–1.41) and ICU discharge (HR 1.63, 95% CI, 1.38–1.92), which suggested that sufentanil was associated with less time to extubation and ICU discharge. There were no significant differences between fentanyl and sufentanil regarding hazards for ventilator mortality (HR 0.95, 95% CI, 0.77–1.17) and ICU mortality (HR 1.03, 95% CI, 0.84–1.26).

Remifentanil was associated with higher hazards for extubation (HR 1.60, 95% CI, 1.40–1.84) and ICU discharge (HR 2.02, 95% CI, 1.43–2.84), relative to fentanyl. However, no significant differences between fentanyl and sufentanil were found for ventilator mortality (HR 0.87, 95% CI, 0.50–1.49) and ICU mortality (HR 1.25, 95% CI, 0.73–2.16). No significant differences were observed between sufentanil and remifentanil regarding hazards for extubation (HR 1.14, 95% CI, 0.92–1.41), ventilator mortality (HR 0.91, 95% CI, 0.47–1.76), ICU discharge (HR 1.31, 95% CI, 0.81–2.14), and ICU mortality (HR 0.66, 95% CI, 0.34–1.28).

### Subgroup Analyses and Sensitivity Analyses

Figures 3, 4 showed the subgroup analyses by surgery. The hazard ratios were similar across the subgroups (test of interaction, *p* > 0.05). Compared to fentanyl, sufentanil was associated with increased hazards for extubation and ICU discharge both in patients with and without surgery. Similarly, compared to patients receiving fentanyl, patients treated with remifentanil had higher hazard ratios for extubation and ICU discharge both among patients with and without surgery.

Sensitivity analyses using the traditional Cox model with time-varying covariates and excluding patient treated with non-opioid analgesics did not show change in interpretation. In the comparisons of sufentanil versus remifentanil, however, the sensitivity analyses using complete cases without imputation generated hazard ratios that excluded the null for extubation (HR 1.36, 95% CI, 1.03–1.80), ICU discharge (HR 2.05, 95% CI, 1.07–3.93), and ICU mortality (HR 0.44, 95% CI, 0.22–0.88) (See Supplementary Table S2.).

## Discussion

In this large observational study, we assessed the association of fentanyl, sufentanil, and remifentanil with important clinical outcomes under the real-world conditions. Our findings showed that treatment of opioids varied during MV treatment in routine practice. Compared to fentanyl, use of sufentanil or remifentanil was associated with shorter time to extubation and ICU discharge; no significant differences in duration of MV and length of ICU stay were found between sufentanil and remifentanil. No significant differences were observed between any of these agents in ventilator mortality and ICU mortality. Subgroup analyses suggested that the effects were similar among patients with and without receiving surgery.

The findings may be due to pharmacokinetic and pharmacodynamic differences of these agents. As important safety concerns, the accumulation of opioids may cause respiratory depression, sedation, immunosuppression, and ileus (Smith et al., 2014; Lee et al., 2015; Gomes et al., 2017). Studies suggested that opioids with short half-life may enable greater titration accuracy with limited drug accumulation (Futier et al., 2012). Compared to fentanyl, the half-life of sufentanil and remifentanil was shorter, which may have potential advantages in reducing accumulation in organs and further result in shortening duration of ventilation and length of ICU stay (Glass et al., 1999; Breen et al., 2004; Rozendaal et al., 2009). However, no differences were observed between sufentanil and remifentanil. This may be because sufentanil is approximately 5–10 times more potent than remifentanil. Due to acute tolerance and opioid-induced hyperalgesia, more pain was reported in the remifentanil group (Welzing et al., 2013; Fletcher and Martinez, 2014; Bhavsar et al., 2016). The adverse effect caused by pain may counteract the effect of limited accumulation in organs.

In our study, the study setting was more complex than RCTs. Nevertheless, the findings under real-world conditions were consistent with those from previous RCTs—our study found that remifentanil may shorten duration of MV and length of ICU stay when compared to fentanyl (Breen et al., 2005; Rozendaal et al., 2009). Our study also suggested that sufentanil and remifentanil may be similar in their clinical effects, consistent with a previous trial (Bhavsar et al., 2016).

Our study also extended the knowledge about these agents in their use for MV patients, which was not identified from previous RCTs. In particular, there were just few studies comparing sufentanil and fentanyl in mechanically ventilated patients. We found that sufentanil was associated with less time to extubation and ICU discharge than fentanyl. A small double-blind RCT, including 20 newborns receiving MV for more than 24 h, suggested that compared to fentanyl, sufentanil did not reduce the weaning time (Schmidt et al., 2010). However, this study exclusively included newborns and had a very small sample size.

### Strengths and Limitations

In this study, we utilized a large database with high level of quality and comprehensiveness of information. The sample size of this study was relatively large. We considered day-to-day changes in opioid exposures and used statistical methods to handle time-dependent variates. We also measured the competing risks and adjusted for an extensive array of potential confounding factors.

However, our study is tempered by important limitations. First, owing to the observational nature, residual confounding from unknown and unmeasured variables is possible, especially for confounding by indication. Prescription of opioid was not randomly performed but determined by clinicians. Although we consider and adjusted for an extensive array of potential confounding, confounding by indication may not be excluded. Second, relatively few patients were exposed to remifentanil, and the inference on the effect of remifentanil is weakened. Finally, this study was conducted exclusively based on a database from a homogenous healthcare system, which may limit the generalization.

In conclusion, our findings suggested that remifentanil was superior to fentanyl in shortening the duration of MV and length of ICU stay, which was consistent with previous RCTs. The effects on ventilator mortality and ICU mortality appeared comparable across these agents. Our finding also showed that sufentanil was associated with less time to extubation and ICU stay, which extends the prior RCT. Further studies are needed to confirm these results.

## Data Availability

The raw data supporting the conclusion of this article will be made available by the authors, without undue reservation.
