# Supplementary material for: Associations of Fentanyl, Sufentanil, and Remifentanil With Length of Stay and Mortality Among Mechanically Ventilated Patients: A Registry-Based Cohort Study
Source: Front Pharmacol. 2022 Mar 4;13:858531. doi: 10.3389/fphar.2022.858531 (PMC8931505; doi:10.3389/fphar.2022.858531)
Supplement: Supplementary file 1 [file Table1.docx]

***Supplementary Material***

**1 Supplementary Table**

**Supplementary Table S1. Treatment of fentanyl and its analogues during ICU stays**

|  | **Fentanyl**  **(n =4778)** | **Sufentanil**  **(n =4008)** | **Remifentanil**  **(n =2233)** |
| --- | --- | --- | --- |
| **Days of administrations (median [IQR])** | 4 [2, 9] | 1 [1, 5] | 1 [1, 1] |
| **Cumulative dose, mg (median [IQR])** | 4.5 [1.5, 10.0] | 0.4 [0.14, 1.1] | 1.0 [1.0, 2.0] |

**Supplementary Table S2. Sensitivity analyses of association between different opioids and outcomes**

|  | **Time to extubation alive** | | **Ventilator mortality** | | **Time to ICU discharge alive** | | **ICU mortality** | |
| --- | --- | --- | --- | --- | --- | --- | --- | --- |
| **Opioids treatment** | **HR (95% CI)** | **P value** | **HR (95% CI)** | **P value** | **HR (95% CI)** | **P value** | **HR (95% CI)** | **P value** |
| **Traditional cox model with time-varying** **covariates** | | | | | | | | |
| Sufentanil vs fentanyl | 1.31 (1.21, 1.42) | <0.001 | 0.99 (0.81, 1.22) | 0.939 | 1.56 (1.33, 1.82) | <0.001 | 0.98 (0.81, 1.19) | 0.855 |
| Refentanil vs fentanyl | 1.62 (1.41, 1.85) | <0.001 | 1.11 (0.65, 1.89) | 0.697 | 2.05 (1.51, 2.80) | <0.001 | 1.03 (0.60, 1.77) | 0.91 |
| Sufentanil vs refentanil | 1.10 (0.89, 1.36) | 0.365 | 0.89 (0.46, 1.73) | 0.74 | 1.07 (0.68, 1.67) | 0.779 | 0.64 (0.34, 1.24) | 0.187 |
| **Complete cases analyses** | | | | | | | | |
| Sufentanil vs fentanyl | 1.26 (1.15, 1.39) | <0.001 | 0.95 (0.75, 1.21) | 0.683 | 1.72 (1.44, 2.05) | <0.001 | 1.01 (0.80, 1.27) | 0.934 |
| Refentanil vs fentanyl | 1.49 (1.26, 1.78) | <0.001 | 1.11 (0.62, 2.00) | 0.721 | 1.63 (1.04, 2.55) | 0.035 | 1.50 (0.82, 2.77) | 0.189 |
| Sufentanil vs refentanil | 1.36 (1.03, 1.80) | 0.031 | 0.65 (0.32, 1.31) | 0.229 | 2.05 (1.07, 3.93) | 0.032 | 0.44 (0.22, 0.88) | 0.021 |
| **Excluding patients receiving nonopioid analgesics** | | | | | | | | |
| Sufentanil vs fentanyl | 1.26 (1.16, 1.38) | <0.001 | 0.99 (0.80, 1.23) | 0.944 | 1.53 (1.29, 1.82) | <0.001 | 1.07 (0.88, 1.31) | 0.501 |
| Refentanil vs fentanyl | 1.63 (1.4, 1.89) | <0.001 | 0.85 (0.48, 1.48) | 0.559 | 1.67 (1.15, 2.44) | 0.008 | 1.26 (0.72, 2.20) | 0.426 |
| Sufentanil vs refentanil | 1.07 (0.85, 1.34) | 0.562 | 0.93 (0.48, 1.79) | 0.817 | 1.36 (0.83, 2.24) | 0.222 | 0.73 (0.37, 1.47) | 0.381 |

Abbreviations: ICU, intensive care unit; HR, hazard ratio;
